# A Novel Method for Floxed Gene Manipulation Using TAT-Cre Recombinase in Ex Vivo Precision-Cut Lung Slices (PCLS)

**DOI:** 10.21769/BioProtoc.4980

**Published:** 2024-04-20

**Authors:** Sek-Shir Cheong, Tiago C. Luis, Matthew Hind, Charlotte H. Dean

**Affiliations:** 1 National Heart and Lung Institute (NHLI), Imperial College London, London, UK; 2 Centre for Inflammatory Diseases, Department of Immunology and Inflammation, Imperial College London, London, UK; 3 National Institute for Health Research (NIHR) Respiratory Biomedical Research Unit, Royal Brompton & Harefield NHS Foundation Trust, London, UK

**Keywords:** Precision-cut lung slices, PCLS, TAT-Cre recombinase, Gene modification, Floxed allele modification, Ex vivo model, TReATS, Permanent gene manipulation, Gene deletion, Gene activation

## Abstract

Precision-cut lung slices (PCLS), ex vivo 3D lung tissue models, have been widely used for various applications in lung research. PCLS serve as an excellent intermediary between in vitro and in vivo models because they retain all resident cell types within their natural niche while preserving the extracellular matrix environment. This protocol describes the TReATS (TAT-Cre recombinase-mediated floxed allele modification in tissue slices) method that enables rapid and efficient gene modification in PCLS derived from adult floxed animals. Here, we present detailed protocols for the TReATS method, consisting of two simple steps: PCLS generation and incubation in a TAT-Cre recombinase solution. Subsequent validation of gene modification involves live staining and imaging of PCLS, quantitative real-time PCR, and cell viability assessment. This four-day protocol eliminates the need for complex Cre-breeding, circumvents issues with premature lethality related to gene mutation, and significantly reduces the use of animals. The TReATS method offers a simple and reproducible solution for gene modification in complex ex vivo tissue-based models, accelerating the study of gene function, disease mechanisms, and the discovery of drug targets.

Key features

• Achieve permanent ex vivo gene modifications in complex tissue-based models within four days.

• Highly adaptable gene modification method that can be applied to induce gene deletion or activation.

• Allows simple Cre dosage testing in a controlled ex vivo setting with the advantage of using PCLS generated from the same animal as *true controls*.

• With optimisation, this method can be applied to precision-cut tissue slices of other organs.

**Graphical overview**


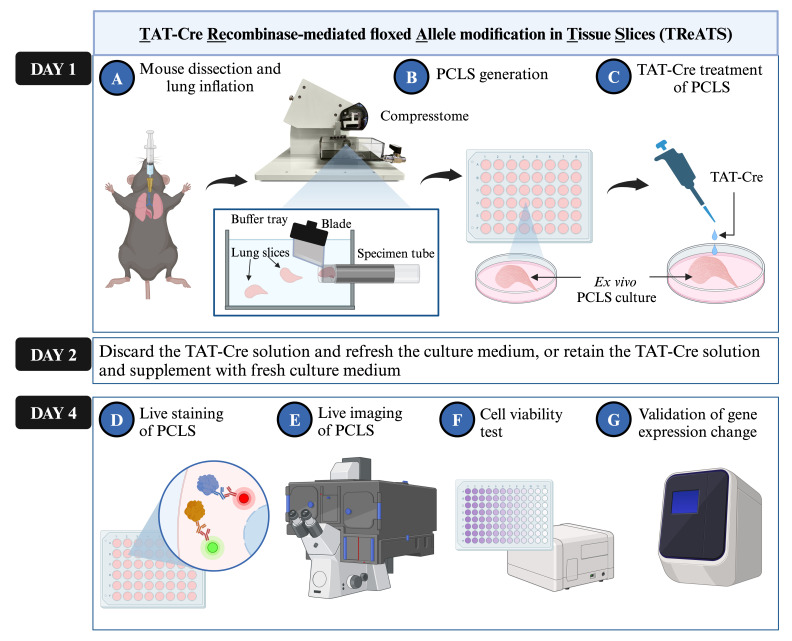


**Workflow of TAT-Cre recombinase-mediated floxed allele modification in tissue slices (TReATS) and validation protocols of gene modification.**

## Background

Precision-cut lung slices (PCLS) represent a 3D ex vivo platform that plays a crucial role in advancing respiratory research. The significance of PCLS lies in their ability to serve as an intermediate model bridging conventional in vitro models and in vivo studies. PCLS preserve the spatial and cellular complexities of the native lung microenvironment while mitigating challenges associated with in vivo experiments, such as ethical considerations, time constraints, and cost limitations [1]. As an ex vivo alternative, PCLS provide a controlled and physiologically relevant setting to investigate various aspects of lung biology, from responses to injury to the behaviour of specific cell types, all within the context of the native lung architecture [2–5]. This advances our understanding of the nuanced interplay between cellular and molecular components governing respiratory function.

Despite the versatility of PCLS, a notable gap has remained to enable effective and permanent gene manipulation in tissue slices, primarily due to the challenges associated with the inherent complexity of the tissue and the imperative to maintain tissue viability. Addressing this gap, our protocol describes a novel approach termed TReATS (TAT-Cre recombinase-mediated floxed allele modification in tissue slices). The TReATS method utilises TAT-Cre recombinase, a cell-permeant Cre protein, to effectively induce ex vivo genetic modifications in PCLS derived from floxed animals, resulting in permanent gene activation or deletion within a four-day timeframe [6]. This simple and effective technique circumvents the need for a Cre-containing mouse allele, eliminating the necessity for complex breeding strategies to generate transgenic mice of interest, thereby remarkedly reducing the time and costs involved and, most importantly, aligning with the 3Rs principles (Refinement, Reduction, and Replacement) by minimising the use of animals in research.

Application of the TReATS method in adult PCLS has important implications for genetic studies, as it overcomes issues related to embryonic or perinatal lethality that can occur upon early gene ablation or overexpression, thereby enabling the possibility to investigate gene function in adulthood [6]. While the TReATS method is useful for inducing ubiquitous pan-tissue gene manipulation across PCLS, its current limitation lies in its inability to induce cell type–specific gene modification. This can only be achieved by crossing with animals possessing specific Cre drivers.

The TReATS method is versatile and adaptable, as evidenced in the original manuscript, where the method is successfully applied to different *loxP*-modified alleles, inducing gene deletion or activation [6]. This adaptability holds great promise for accelerating the discovery of gene function, unravelling disease mechanisms, and exploring potential therapeutic interventions.

## Materials and reagents

**Biological materials**

1. *Rosa26-flox-stop-flox-EYFP* mouse (10–12 weeks; male/female) (from Dr. Tiago Luis; strain: 006148; RRID: IMSR_JAX:006148). Abbreviation: *R26R-EYFP*
2. Wildtype C57BL/6J mouse (10–12 weeks; male/female; serves as a negative control) (Charles River, catalog number: 632)

**Reagents**

1. Dulbecco’s modified Eagle medium (DMEM-Glutamax) (Sigma-Aldrich, catalog number: 31966021)
2. Phenol red–free DMEM (Life Technologies, catalog number: 21063029)
3. Low-gelling temperature agarose (Sigma-Aldrich, catalog number: A9414)
4. Hank's balanced salt solution (HBSS) (Sigma-Aldrich, catalog number: H6648)
5. 4-(2-hydroxyethyl)-1-piperazineethanesulfonic acid (HEPES) buffer (Life Technologies, catalog number: 15630080)
6. Penicillin-streptomycin 10,000 U/mL (Merck Life Science UK, catalog number: P0781)
7. Phosphate buffered saline (PBS) (Life Technologies, catalog number: 10010056)
8. Super glue liquid precision bottle 5 g (Loctite, catalog number: 2632836)
9. TAT-Cre recombinase (Merck, catalog number: SCR508)
10. Prolong^TM^ live antifade reagent (Invitrogen, catalog number: P36975)
11. Cell proliferation kit (MTT assay) (Roche, catalog number: 11465007001)
12. Dimethyl sulfoxide (DMSO) (Merck Life Science UK, catalog number: D5879)
13. Absolute ethanol for molecular biology (Sigma-Aldrich, catalog number: 51976)
14. Methanol (Sigma-Aldrich, catalog number: 34860)
15. RNase Zap RNase decontamination solution (Life Technologies, catalog number: AM9780)
16. Ultrapure DNase/RNase-free distilled water (Life Technologies, catalog number: 10977035)
17. RNeasy mini kit (Qiagen, catalog number: 74104)
18. RNase-free DNase set (Qiagen, catalog number: 79254)
19. RNA screen tape (Agilent, catalog number: 5067-5576)
20. RNA screen tape sample buffer (Agilent, catalog number: 5067-5577)
21. High-capacity cDNA reverse transcription kit (Applied Biosystems, catalog number: 4368814)
22. TaqMan fast advanced master mix (Life Technologies, catalog number: 4444556)
23. (Optional) *Mouse B2m* gene expression assay (Life Technologies, catalog number: 4331182_Mm00437762_m1)
24. (Optional) *YFP* gene expression assay (Life Technologies, catalog number: 4331182_ Mr04097229_mr)

**Solutions**

1. HBSS/HEPES buffer (see Recipes)
2. Agarose solution 2% (w/v) (see Recipes)
3. Serum-free (SF)-DMEM culture medium (see Recipes)
4. Imaging medium (see Recipes)
5. MTT working solution (see Recipes)
6. 70% (v/v) methanol (see Recipes)
7. 70% (v/v) ethanol (see Recipes)
8. RLT/β-mercaptoethanol lysis buffer (see Recipes)

**Recipes**

1. **HBSS/HEPES buffer (store at 4 °C for up to six months)**
  **Table:** 

Reagent	Final concentration	Volume
HBSS	n/a	495 mL
HEPES	10 mM	5 mL
Total	n/a	500 mL
2. **Agarose solution 2% (w/v) (freshly prepared and used)**
  **Table:** 

Reagent	Final concentration	Quantity
HBSS/HEPES	n/a	50 mL
Agarose	2% (w/v)	1 g
Total	n/a	50 mL
3. **SF-DMEM culture medium (store at 4 °C for up to six months)**
  **Table:** 

Reagent	Final concentration	Volume
DMEM-Glutamax	n/a	495 mL
Penicillin-streptomycin	100 U/mL	5 mL
Total	n/a	500 mL
4. **Imaging medium (freshly prepared and used)**
  **Table:** 

Reagent	Final concentration	Volume
Phenol red–free DMEM	n/a	49 mL
Penicillin-streptomycin	100 U/mL	500 µL
Prolong^TM ^live antifade reagent	1% (v/v)	500 µL
Total	n/a	50 mL
5. **MTT working solution (freshly prepared and used)**
  **Table:** 

Reagent	Final concentration	Volume
SF-DMEM	n/a	2.7 mL
MTT solution (Cell proliferation kit)	10% (v/v)	300 µL
Total	n/a	3 mL
6. **70% methanol (store at room temperature)**
  **Table:** 

Reagent	Final concentration	Volume
Methanol	70% (v/v)	14 mL
H_2_O	n/a	6 mL
Total	n/a	20 mL
7. **70% ethanol (store at room temperature)**
  **Table:** 

Reagent	Final concentration	Volume
Absolute ethanol	70% (v/v)	14 mL
H_2_O	n/a	6 mL
Total	n/a	20 mL
8. **RLT/β-mercaptoethanol lysis buffer (freshly prepared and used)**
  **Table:** 

Reagent	Final concentration	Amount
RLT buffer (RNeasy mini kit)	n/a	1,980 µL
β-mercaptoethanol	1% (v/v)	20 µL
Total	n/a	2 mL

**Antibodies and fluorescent dyes**

1. Alexa Fluor^®^ 647-conjugated rat anti-lysosomal associated membrane protein 3 (LAMP3) (marker for mature differentiated alveolar type II (ATII) cells) (Dendritics, catalog number: DDX0192A647), dilution: 1:250
2. Podoplanin eFluor^®^ 660 [alveolar type I (ATI) cell marker] (Life Technologies, catalog number: 50-5381-82), dilution: 1:500
3. Alexa Fluor^®^ 647-conjugated platelet endothelial cell adhesion molecule (PECAM) (vascular endothelium marker) (BioLegend, catalog number: 102416), dilution: 1:200
4. Alexa Fluor^®^ 647-conjugated CD11c (macrophage marker) (BioLegend, catalog number: 117312), dilution: 1:200
5. BioTracker^TM^ TiY vimentin live cell dye (fibroblast marker) (Sigma-Aldrich, catalog number: SCT059), dilution: 1:2,000
6. Armenian hamster IgG-PE isotype control (BioLegend, catalog number: 400907), dilution: 1:200
7. Rat IgG-Alexa Fluor 647 isotype control (BioLegend, catalog number: 400526), dilution: 1:200
8. 4′,6-diamidino-2-phenylindole (DAPI) 1 mg/mL (Life Technologies, catalog number: 62248), dilution: 1:500

**Laboratory supplies**

1. 48-well clear TC-treated culture plate (Scientific Laboratory Supplies Ltd., catalog number: 3548)
2. 96-well clear flat bottom TC-treated culture plate (Scientific Laboratory Supplies Ltd., catalog number: 3596)
3. 24-well uncoated µ-plates (Ibidi, catalog number: 82421)
4. Hydrophilic PTFE 12 mm cell culture inserts with 0.4 µm pores (Millipore, catalog number: PICM01250)
5. Costar 5 mL stripette serological pipets (Scientific Laboratory Supplies Ltd., catalog number: 4487)
6. Costar 10 mL stripette serological pipets (Scientific Laboratory Supplies Ltd., catalog number: 4488)
7. Costar 25 mL stripette serological pipets (Scientific Laboratory Supplies Ltd., catalog number: 4489)
8. Syringe 5 mL luer (VWR, catalog number: 613-2042)
9. Swann-Morton surgical scalpels, No. 22 (Fisher Scientific Ltd., catalog number: 11758353)
10. Sterile bijou 7 mL (Greiner Bio-One Ltd, catalog number: 189171)
11. Weigh boats square 7 mL white (VWR, catalog number: 611-0093)
12. Qualfast Zinc-plated steel washer M6 (Cromwell, catalog number: QFT6455541N)
13. Petri dishes, 90 mm × 16 mm (Sarstedt, catalog number: 82.1472)
14. 0.2 mL 8-strip non-flex PCR tubes (Starlab, catalog number: I1402-3700)
15. Homogenisation tubes, screw cap micro tube 2 mL (Sarstedt, catalog number: 72.609)
16. Homogenisation tube caps, standard screw cap (Starlab, catalog number: E1480-0100)
17. Homogenisation beads, fastPrep-24^TM^ lysing matrix D 1.4 mm ceramic spheres (MP Biomedicals, catalog number: 1169131050-CF)
18. TapeStation loading tips (Agilent, catalog number: 5067-5153)
19. TapeStation sample tube strips (Agilent, catalog number: 4014128)
20. TapeStation sample tube caps (Agilent, catalog number: 4014125)
21. MicroAmp^TM^ fast optical 96-well reaction plate (ABI Applied Biosystems, catalog number: 4346907)
22. MicroAmp^TM^ optical adhesive film (ABI Applied Biosystems, catalog number: 4311971)
23. Ice and ice box
24. Laboratory tissue roll

## Equipment

1. Automated compresstome (Precisionary Instruments, catalog number: VF-510-0Z)
2. Stainless steel blades for vibratome (Campden Instruments LTD, catalog number: 7550-1-SS)
3. Leica SP8 inverted confocal microscope (Leica)
4. SpectraMax^®^ iD3 microplate reader (Molecular Devices, model: iD3, serial number: 37370-3811)
5. FastPrep-24^TM^ classic bead tissue homogeniser (MP Biomedicals, catalog number: 116004500)
6. TapeStation 2200 (Agilent, catalog number: 18588)
7. VeritiPro^TM^ thermal cycler (Applied Biosystems, catalog number: A48141)
8. StepOnePlus^TM^ real-time PCR system (Applied Biosystems, catalog number: 4376600)
9. NanoDrop spectrophotometer (Thermo Scientific, catalog number: ND-1000)
-20 °C freezer
11. Incubator (humidified, 37 °C, 5% CO_2_) (Sanyo, catalog number: MCO-17A)
12. Prism benchtop microfuge (Appleton Woods, catalog number: AA9760)
13. Centrifuge vortex for PCR plate CVP-2 (Grant Bio, catalog number: CVP-2)
14. IKA MS 3 basic shaker with MS 3.5 PCR plate attachment (IKA, catalog number: 0003617000)
15. Curved dissecting forceps, 10 cm, Serr/C (World Precision Instruments Ltd, catalog number: 15915)
16. Dissecting scissors, 10 cm, straight (World Precision Instruments Ltd, catalog number: 14393)
17. Surgical scissors, 14 cm, straight (World Precision Instruments Ltd, catalog number: 14192)
18. Spring scissors, 12 cm straight, 12 mm extra-fine blades (World Precision Instruments Ltd, catalog number: 14125)
19. Metallic spatula (Fisher Scientific Ltd., catalog number: 11523482)

## Software and datasets

1. Leica Application Suite X software (Leica)
2. Fiji (ImageJ, version 2.9)
3. GraphPad Prism 10 (GraphPad)
4. 2200 TapeStation System (Agilent, G2964AA)
5. StepOne Software (Applied Biosystems)

## Procedure

- **Mouse dissection and lung inflation**
  Perform mouse dissection and lung inflation as previously described in Procedure A1 “Mouse dissection and lung harvesting” (refer to Video 1 and Figure 1) [7].
- **PCLS generation**
  Generate PCLS as previously described [7], with slight modifications (Video 1).
  **Video 1.**
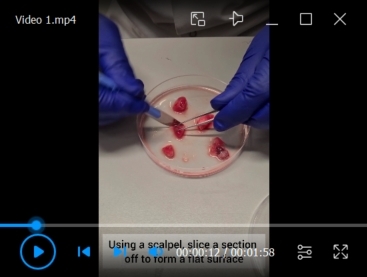
Precision-cut lung slices (PCLS) generation using compresstome
  1. Preparation
    - Cool the chilling block in the -20 °C freezer for 1 h before use.
    - Attach the stainless-steel blade to the blade holder using super glue. Use a paper clip to hold it in place and air dry at room temperature for 10 min before use.
    - Prepare 200 mL of HBSS/HEPES buffer (see Recipes) and a 48-well plate with ice-cold SF-DMEM (300 μL/well) and keep on ice until use.
    - Disinfect the surgical tools, spatula, specimen holder, and the buffer tray of the compresstome by wiping them with 70% ethanol (see Recipes). Let them air dry.
    - Prepare 50 mL of 2% (w/v) agarose solution (see Recipes) and an icebox with lukewarm water to keep the agarose warm and prevent it from solidifying throughout the slicing process.
  2. Precision-cut lung slicing
    - In a 90 mm Petri dish, separate the fresh lung lobes using forceps and dissecting scissors.
    - Slice a tiny section off from the basal end of the tissue to form a flat surface using a scalpel blade. This ensures that the tissue can be securely glued to the specimen tube.
    - Squeeze a tiny drop of super glue onto the specimen tube base.
*Note: Do not use too much super glue, as this will cause the specimen tube plunger to stick to the metal tube.*
    - Place the lung lobe vertically onto the glue using forceps and allow the super glue to cure for 1 min.
    - Pull the specimen tube plunger downwards until the tissue is fully covered by the metal tube.
    - Use a 5 mL syringe and pipette enough lukewarm agarose solution to cover the tissue and ensure no bubbles formed around it.
    - Place the chilling block around the specimen tube for 1 min to solidify the agarose.
    - Once the agarose is solidified (turns from transparent to translucent), insert the specimen tube into the buffer tray fully until the stopper ring touches the adapter.
    - Adjust the micrometer until it touches the back of the specimen tube.
    - Slide the blade holder into the axial bar of the vibrating unit and secure it with the Allen key.
    - Add ice-cold HBSS/HEPES buffer into the buffer tray until the specimen tube is submerged in the buffer.
    - Set the compresstome to start slicing at a thickness of 300 μm, advance speed 4, and oscillation 4.
    - The desired thickness is 250 μm. Start slicing on continuous mode at 300 μm and reduce the thickness by 10 μm after each slice is produced (300 μm → 290 μm → 280 μm → 270 μm → 260 μm → 250 μm) until reaching 250 μm. Continue slicing at the thickness of 250 μm until the entire embedded tissue is completely sliced.
*Note: The gradual reduction in thickness helps to produce slices with consistent and accurate thickness.*
    - Collect the PCLS with a metal spatula and place them into the 48-well plate containing 300 μL of SF-DMEM (one PCLS per well) as the slicing progresses.
*Note: As the number of live cells is a critical factor that determines the metabolic activity in MTT assays, it is recommended to use PCLS generated from the middle of the lung lobe to ensure size conformity.*
    - Incubate the PCLS in the incubator (37 °C, 5% CO_2_) for 2 h before proceeding with the washing steps.
    - After incubation, wash the PCLS with 300 μL of prewarmed SF-DMEM per well. Incubate at 37 °C in 5% CO_2 _for 5 min during each wash and repeat the washing step three times.
**Pause point:** The PCLS can be incubated overnight at 37 °C in 5% CO_2 _before proceeding with the washing steps the next day. This does not significantly affect the efficacy of TAT-Cre treatment.
- **TAT-Cre treatment of PCLS**
  1. Experimental controls:
    - Negative control for Cre protein
      Treat PCLS from the same *R26R-EYFP* mouse with only SF-DMEM. These PCLS serve as the negative control to exclude leaky transgene.
    - Control for artefacts due to the TAT-Cre treatment
      Use PCLS from a wildtype mouse of the same background strain as the transgenic mouse as a control to exclude artefacts caused by the TAT-Cre treatment.
  2. TAT-Cre recombinase treatment
    - After the washing steps, add 3 μM of TAT-Cre recombinase solution diluted in prewarmed SF-DMEM to the *R26R-EYFP* and wildtype mouse PCLS (250 μL/well) and incubate for 24 h at 37 °C in 5% CO_2_ (Figure 1A). Untreated *R26R-EYFP* PCLS with SF-DMEM are used as negative controls.
    - After 24 h of incubation, remove the TAT-Cre solution. Add fresh prewarmed SF-DMEM into the PCLS (250 μL/well) and incubate at 37 °C in 5% CO_2 _for a further 48 h.
      See Troubleshooting.
    **Figure 1.**
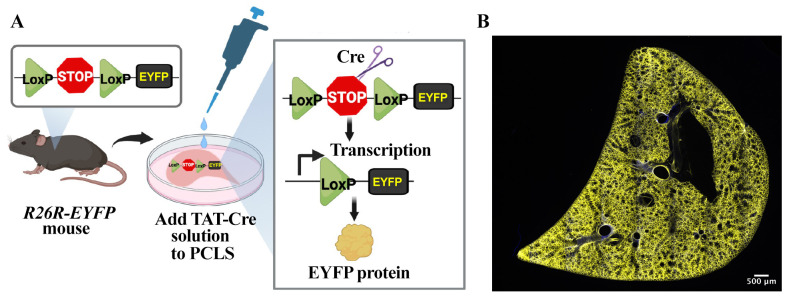
TReATS method activates the expression of *EYFP* transgene in precision-cut lung slices (PCLS) generated from *R26R-EYFP* mouse. (A) Schematic showing a simplified structure of the *loxP*-modified allele in *R26R-EYFP* mouse and the structure of the targeted locus after Cre-mediated excision of the *loxP*-flanked stop sequence. TAT-Cre recombinase-mediated excision of the upstream *loxP*-flanked stop sequence allows the transcription and translation of *EYFP* transgene. (B) Tiled image shows ubiquitous expression of EYFP protein in TAT-Cre-treated *R26R-EYFP* PCLS. Image was generated using a confocal microscope.
- **Live staining of PCLS**
  1. To visualise in which alveolar cell types the TAT-Cre recombinase-mediated EYFP activation occurs (Figure 1B), at 72 h post-Cre treatment, immunolabel key alveolar cell type markers with fluorescent dye-conjugated antibodies, as listed in Materials and Reagents.
  2. Incubate the PCLS with Alexa Fluor^®^ 647-conjugated rat anti-LAMP3 (1:250), podoplanin eFluor^®^ 660 (1:500), Alexa Fluor^®^ 647-conjugated PECAM (1:200), Alexa Fluor^®^ 647-conjugated CD11c (1:200), or BioTracker^TM^ TiY vimentin live cell dye (1:2,000) diluted in prewarmed SF-DMEM for 2 h at 37 °C in the dark. Use 250 μL of diluted antibody per PCLS per well in a 48-well plate.
  3. To validate the absence of any non-specific binding of the conjugated fluorophores, stain PCLS with appropriate isotype IgG control antibodies diluted in prewarmed SF-DMEM for 2h at 37 °C in the dark: Armenian hamster IgG-PE (1:200) for TiY vimentin or rat IgG-Alexa Fluor 647 (1:200) for LAMP3, PDPN, PECAM, and CD11c. Use 250 μL of diluted antibody per PCLS per well in a 48-well plate.
  4. After incubation, discard the culture medium with antibodies and add 250 μL of DAPI (1:500) diluted in prewarmed SF-DMEM for 15 min at 37 °C in the dark to counterstain the cell nuclei.
  5. Remove DAPI, wash PCLS three times with 300 μL of prewarmed PBS, and immediately set up the PCLS for live imaging.
*Note: It is recommended to image the immunostained PCLS on the same day to avoid fluorophore signal loss.*
- **Live imaging of PCLS using confocal microscope**
  1. Pre-equilibrate the incubator chamber of an inverted confocal microscope with the following conditions: 37 °C, 5% CO_2_, and room air oxygen levels, approximately 21%, for 30 min.
*Note: It is crucial to equilibrate the incubator chamber before imaging to maintain the viability of PCLS throughout the imaging process, particularly if the imaging is expected to take over an hour.*
  2. Meanwhile, add 100 μL of prewarmed imaging medium (see Recipes) into each well in an Ibidi uncoated 24-well µ-plate. Carefully transfer the immunostained PCLS from the 48-well plate onto the imaging medium using a metal spatula.
  3. Using forceps, gently place a 12 mm Millicell^®^ cell culture insert with 0.4 μm pores onto the PCLS so that the PCLS is positioned at the centre of the insert. Secure the insert in place by placing a flat metal washer on top of the insert (Figure 2A).
  4. Add 200 μL and 400 μL of imaging media into the bottom chamber and upper chamber, respectively. Cover the 24-well plate with a lid.
  5. Once the incubator chamber is equilibrated, place the Ibidi 24-well plate containing PCLS onto the motorised stage. Image using an HC PL APO 10×/0.40 air objective lens (Figure 2B, 2C) or HC PL APO 40×/1.30 oil objective lens (Figure 2D–2H). Use the following lasers: Diode 405 nm (for DAPI), argon 514 (for EYFP), Diode 561 (for vimentin), and Diode 633 (for LAMP3, PDPN, PECAM, and Cd11c) (Figure 2D–2H).
*Note: Low magnification (10× objective lens) is recommended for an overview of EYFP activation across the entire PCLS; high magnification (40× objective lens) is recommended to visualise co-localisation of EYFP with different alveolar cell type markers.*
    **Figure 2.**
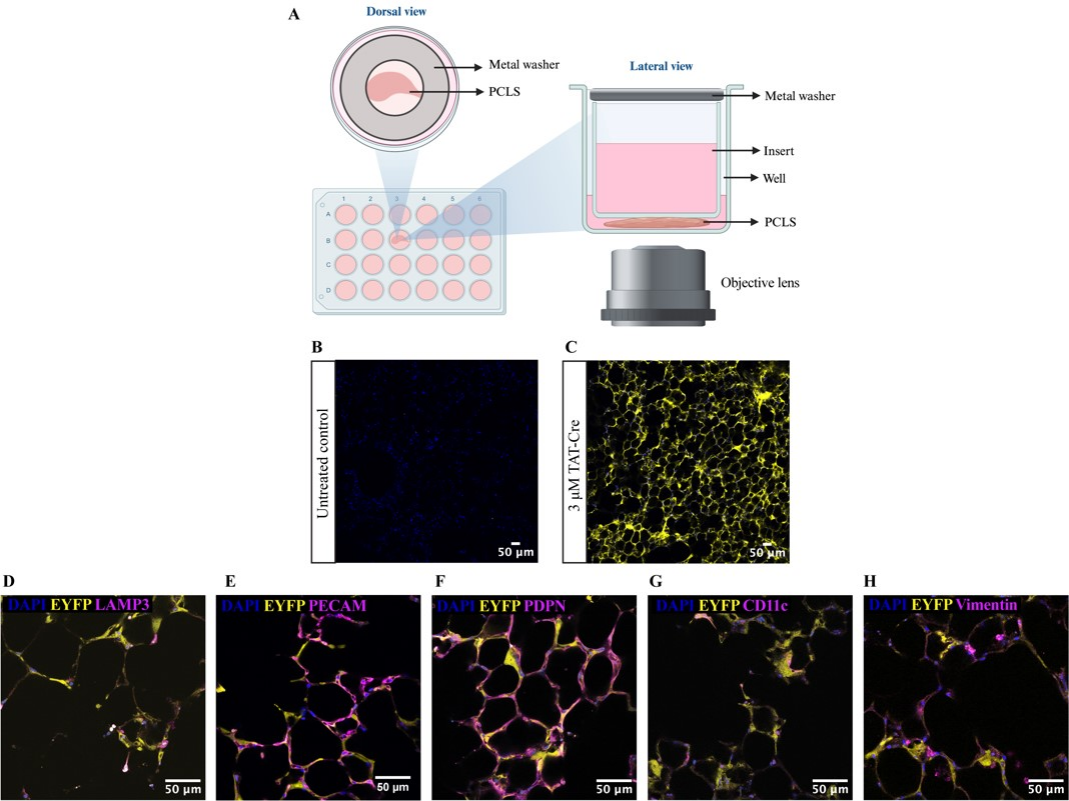
Live imaging of precision-cut lung slices (PCLS) stained with specific cell type markers. (A) Schematic diagram shows the live imaging setup that utilises a transwell insert and a metal washer to secure the PCLS in place throughout the imaging process. (B-C) Representative images showing untreated *R26R-EYFP* PCLS (B) and TAT-Cre recombinase-treated *R26R-EYFP* PCLS (C). EYFP protein expression is shown in yellow and cell nuclei were labelled with DAPI (blue). Images were captured on a confocal microscope using an HC PL APO 10×/0.40 air objective lens. (D–H) Images showing co-localisation of EYFP with different alveolar cell type markers: LAMP3 (mature ATII cells) (D), PECAM for endothelial cells (E), PDPN for ATI cells (F), CD11c for macrophages (G), and vimentin for fibroblasts (H). Images were taken using 40× objective lens on a confocal microscope.
  6. Image five random fields of alveolar regions per PCLS at approximately 40 μm from the bottom of the PCLS.
*Note: A distance of 40 μm from the bottom of the PCLS is recommended as the PCLS are generally at the best focus at this distance. Imaging further from this distance is possible but can become challenging due to limited light penetrance through the depth of PCLS. To image the whole depth of PCLS, Z-stack is required, and this has been described in the original manuscript. It is also recommended to avoid imaging the PCLS less than 20 μm from the bottom of the PCLS, as the slicing process injures the surface of the PCLS, and an intact lung architecture may not be retained closer to the sliced surface. Thus, imaging at 40 μm from the bottom of the PCLS provides a convenient and representative snapshot of the PCLS.*
- **Cell viability test (MTT assay)**
  1. At 72 h post-Cre treatment, perform MTT assays to examine whether the Cre treatment affects cell viability by assessing cell metabolic activities within PCLS.
*Note: Use PCLS of equal size generated from the middle of the lung lobe for MTT assays.*
  2. Prepare 10% MTT solution (see Recipes). Use 250 μL of 10% MTT solution for each PCLS per well in a 48-well plate.
  3. Positive control for dead cells: PCLS treated with 70% methanol serve as control for dead cells. Add 250 μL of 70% methanol (see Recipes) into the control PCLS and incubate at room temperature for 15 min. After 15 min, remove the methanol solution and wash the PCLS three times with 300 μL of room-temperature PBS. Proceed with step F4.
  4. Remove the culture medium. Add 250 μL of 10% MTT solution into the PCLS and incubate at 37 °C and 5% CO_2_ in the dark for 45 min.
  5. After incubation, discard the MTT solution. Add 250 μL of DMSO into each PCLS and incubate at 37 °C and 5% CO_2_ in the dark for a further 10 min.
  6. After DMSO incubation, gently pipette up and down a few times and transfer 200 μL from each well into a new clear-bottom 96-well plate.
  7. Read the absorbance at 570 nm and 690 nm and calculate cell viability (see Data analysis).
*Note: Use four mice per genotype and three PCLS per treatment per experiment.*
- **Validation of gene expression change**
  1. PCLS homogenisation and RNA extraction
    - Use RNase decontamination solution to clean the working surface.
    - Steps G1b–g are illustrated in Figure 3. In each homogenisation tube, add homogenisation beads to the level of the bottom cone line of the tube. Label each tube with the sample name.
      **Figure 3.**
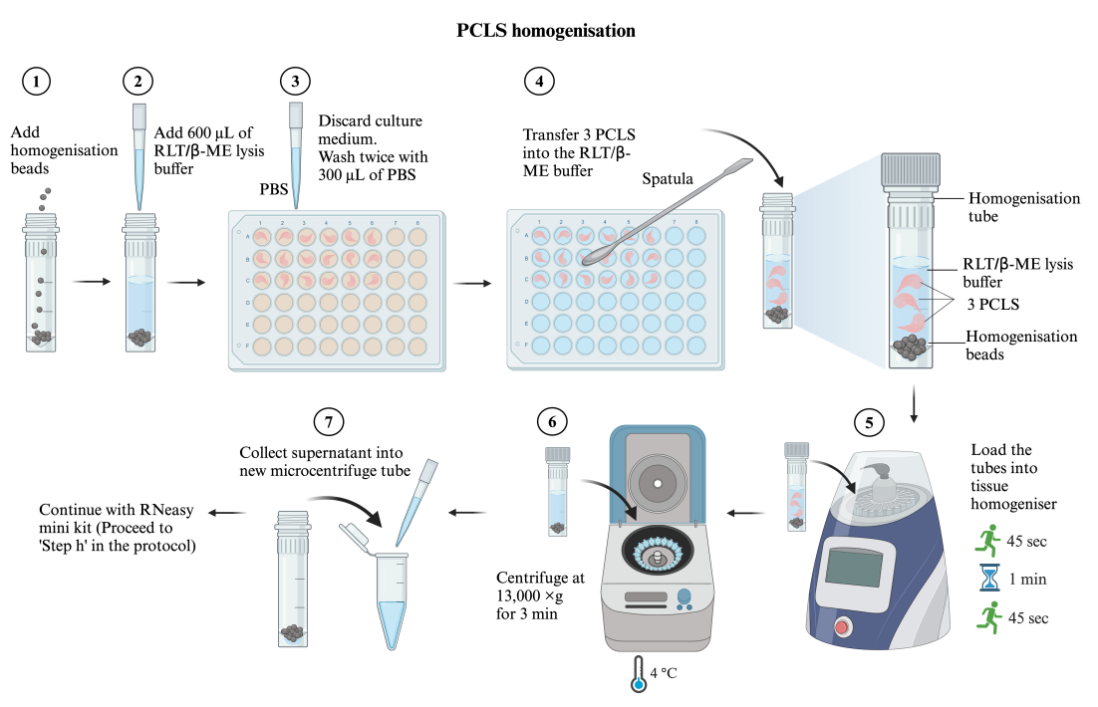
Precision-cut lung slices (PCLS) homogenisation workflow
    - Add 600 μL of RLT/β-ME lysis buffer (see Recipes) into each homogenisation tube.
    - Discard the culture medium and gently wash the PCLS twice with 300 μL of room-temperature PBS.
*Note: It is important to wash the PCLS with PBS. Incomplete removal of culture medium will inhibit lysis and may reduce RNA yield.*
    - Use a metal spatula to transfer the PCLS into the homogenisation tube with RLT/β-ME lysis buffer. Pool three PCLS of the same treatment into each homogenisation tube.
*Note: Pool three PCLS for an RNA sample and three RNA samples for each condition per experiment.*
    - Load the tubes onto the rotor of the FastPrep-24 tissue homogeniser and secure them tightly with the rotor holder. Run the homogeniser for 45 s twice, with a 1-min resting interval.
    - After homogenisation, centrifuge the tubes at 13,000× *g* for 3 min at 4 °C. Collect the supernatant (approximately 600 μL) into a new microcentrifuge tube.
    - Add an equal volume (600 μL) of 70% ethanol (see Recipes) into the PCLS lysate and gently pipette up and down a few times.
    - Transfer 600 μL of the sample into the RNeasy spin columns. Centrifuge at 13,000× *g* at room temperature for 1 min. Discard the flowthrough. Repeat this by transferring the remaining 600 μL of lysate–ethanol mixture into the same RNA columns. Centrifuge again at 13,000× *g* at room temperature for 1 min and discard the flowthrough.
    - Add 350 μL of RW1 washing buffer (provided in the RNeasy mini kit) into each tube. Centrifuge again at 13,000× *g* at room temperature for 1 min and discard the flowthrough.
    - DNase digestion: prepare DNase I solution. For each RNA sample, add 10 μL of DNase I stock solution to 70 μL of RDD buffer (provided in the RNase-free DNase set). Mix by gently inverting the tube and centrifuge briefly.
*Note: Do not vortex as DNase I is particularly sensitive to physical denaturation.*
    - Add 80 μL of DNase I mix directly to the RNeasy spin column and incubate at room temperature for 15 min.
    - After incubation, add 350 μL of RW1 buffer to the column, centrifuge at 13,000× *g* at room temperature for 1 min, and discard the flowthrough.
    - Add 500 μL of RPE buffer to the column, centrifuge at 13,000× *g* at room temperature for 1 min, and discard the flowthrough.
    - Add 500 μL of RPE buffer to the column and centrifuge at 13,000× *g* at room temperature for 2 min.
    - Place the RNeasy spin column into a new collection tube and centrifuge at 13,000× *g* at room temperature for 1 min to remove residual solution.
    - Place the RNeasy spin column into a new collection tube. Add 30 μL of RNase-free water to the spin column and centrifuge at 13,000× *g* for 1 min. To increase the yield of RNA, transfer the eluent back to the spin column and repeat the centrifugation step.
    - Collect the total RNA sample into a new microcentrifuge tube. Store at -80 °C until use.
  2. Assessment of RNA quality and concentration
    - Allow RNA ScreenTape sample buffer to equilibrate at room temperature for 30 min and vortex it before use.
    - Thaw total RNA samples on ice. Mix 5 μL of RNA sample buffer with 1 μL of RNA sample.
    - Spin down and then vortex using IKA vortexer and adaptor at 2,000 rpm for 1 min. Spin down to collect the solution at the bottom of the tube.
    - Denature the samples at 72 °C for 3 min using a thermal cycler, followed by 2 min cooling on ice.
    - Spin down to collect the sample at the bottom of the tube.
    - Load the samples and loading tips into the 2200 TapeStation instrument and start the run.
*Note: Only RNA samples with RNA integrity number (RIN) > 8 are used for downstream experiments.*
  3. cDNA reverse transcription
    - Thaw the kit components on ice and prepare the RT master mix on ice according to Table 1:
      **Table 1.** Reverse transcription reaction setup

Kit component	Volume per reaction (μL)
10× RT buffer	2
25× dNTP mix (100 mM)	0.8
10× RT random primers	2
MultiScribe^TM ^reverse transcriptase	1
RNA sample	200 ng
Top up with nuclease-free H_2_O to 20 μL (per reaction)
    - Pipette the mixture up and down a few times to mix.
    - Close the lids of the tubes and spin down to collect the solution to the bottom of the tubes.
    - Load the samples onto the thermal cycler and start the run using the following protocol: 25 °C for 10 min, 37 °C for 2 h, 85 °C for 5 min, followed by indefinite holding at 4 °C.
    - Store the cDNA samples at -20 °C freezer until use.
  4. Quantitative real-time PCR (qRT-PCR)
    - Thaw cDNA samples on ice and measure the concentration of the cDNA samples using a Nanodrop. Use nuclease-free water as blank.
    - Dilute the cDNA samples to 50 ng/μL using nuclease-free water.
    - Thaw TaqMan^®^ assays on ice.
*Note: Use appropriate target assays accordingly. YFP (Assay ID: Mr04097229_mr) and B2m (Assay ID: Mm00437762_m1; reference gene) assays are used in this protocol (see Reagents).*
    - Mix the Fast Advanced Master Mix by pipetting up and down a few times.
    - Calculate and prepare the qRT-PCR reaction mix in 8-tube PCR strips according to Table 2 (volume per reaction × number of reactions):
      **Table 2.** qRT-PCR reaction setup

Kit component	Volume per reaction (μL)
2× TaqMan^®^ Fast Advanced Master Mix	5
20× TaqMan^®^ Fast gene expression assay	0.5
cDNA template (50 ng/μL)	2
Nuclease-free water	2.5
Total volume per reaction	10

*Note: Prepare three reactions (triplicates) per gene for each cDNA sample.*
    - After all the components are added to the tubes, close the caps and vortex briefly to mix.
    - Briefly centrifuge the 8-tube PCR strips using a benchtop microfuge with 4 × 8 PCR strip rotor to spin down the contents.
    - Pipette 10 μL of the qRT-PCR reaction mix into each well of a 96-well PCR plate.
    - Seal the plate with an optical adhesive film. Centrifuge briefly using a microfuge with a 96-well plate rotor to eliminate the air bubbles.
    - Run the PCR on a StepOne Plus Real-Time PCR System using the following parameters: UNG incubation at 50 °C for 2 min, polymerase activation at 95 °C for 20 s, followed by 40 cycles of (denaturation at 95 °C for 1 s and annealing at 60 °C for 20 s).
    - Analyse the relative transcript levels using the 2^−ΔΔCT^ method (see Data analysis).
*Note: Use four mice per genotype, pool three PCLS per RNA sample, and three RNA samples per treatment per experiment.*

## Data analysis

1. For MTT assays, cell viability is calculated using the following formula:
Cell viability = Mean OD_Adj_ Cre‐treated PCLS/Mean OD_Adj_ control PCLS
  in which,
  OD_Adj_ (Adjusted OD) = OD_570nm_ – OD_690nm_
  Use three PCLS per treatment (triplicates):
Mean OD_Adj_ = OD_Adj_ PCLS_1_ + OD_Adj_ PCLS_2_ + OD_Adj_ PCLS_3_
2. For qRT-PCR, gene expression change is calculated using the 2^-ΔΔCT^ formula, in which
ΔCT =CT_target gene_ – CT_reference gene_
  Pool three PCLS for each RNA sample and prepare three RNA samples per treatment per experiment (triplicates; nine PCLS required per treatment):
Mean ΔCT = ΔCT cDNA_1_ + ΔCT cDNA_2_ + ΔCT cDNA_3_/3
  ΔΔCT = Mean ΔCT_Cre-treated PCLS_ – Mean ΔCT_control PCLS_
  2^-ΔΔCT^ represents the fold change of the treated target gene relative to the control PCLS.
3. Repeat the experiments with a total of four mice (N = 4). Use non-parametric tests to compare the difference between Cre-treated and control PCLS.

## Validation of protocol

This protocol or parts of it has been used and validated in the following research article:

Cheong et al. [6]. A method for TAT-Cre recombinase-mediated floxed allele modification in ex vivo tissue slices. Disease Models & Mechanisms (Figure 1, panels A–E; Figure 2, panels A–E; Movie 1).

## General notes and troubleshooting

1. If the efficacy of TAT-Cre recombination is insufficient, consider increasing the dosage of TAT-Cre and/or extending the incubation time of PCLS in the TAT-Cre solution. Refresh the SF-DMEM every other day if prolonged incubation of PCLS is required. Our experiments found that using up to 5 μM of TAT-Cre and incubating PCLS with the TAT-Cre solution for 72 h did not significantly impact the viability of PCLS.
2. As a reference, a minimum of 15 PCLS from a single animal are required per experimental condition. These include three PCLS for TAT-Cre treatment, three PCLS for MTT assay, and nine PCLS to generate three RNA samples for qRT-PCR analysis. For example, an experiment comparing untreated control PCLS vs. 3 μM TAT-Cre-treated PCLS will require 30 PCLS from a single mouse, and it is recommended to use four mice to confirm reproducibility.
  Optional: If co-localisation staining is required, as described in this protocol, add three PCLS per condition per marker/antibody used. A single PCLS can be labelled with multiple markers/antibodies as long as the fluorophore spectra do not overlap.
3. A single RNA sample generated from pooling three PCLS generally has a yield of 1.5–3.0 μg (concentrations of 50–100 ng/μL in a total volume of 30 μL). The yield varies depending on the size of the PCLS used.
4. To validate the efficiency of the TReATS method, additional techniques beyond qRT-PCR and immunostaining, as described in this protocol, can be utilised. These include PCR to confirm DNA sequence changes as well as western blotting or flow cytometry to assess changes at the protein levels. Additionally, if the effect of gene modification is known, other readouts may also prove useful in validating the efficiency of the TReATS method. For instance, the lack of a functional *Vangl2* gene is known to disrupt actin cytoskeleton remodelling, a phenotype that could be readily detected. In TAT-Cre recombinase-treated *Vangl2^flox/flox^* PCLS, highly disrupted filamentous actin (F-actin) was observed, indicating successful deletion of the *Vangl2* gene in the PCLS. This was described in the original manuscript [6].
